# Did I Take My Medication Today? Understanding Medication Self‐Management for Adults With Intellectual Disabilities Through Participatory Research

**DOI:** 10.1111/jar.70059

**Published:** 2025-04-30

**Authors:** Natasha A. Spassiani, Anna Higgins, Stephan Tait, Aaron Hume, Sam Abdulla, Ruth Paterson

**Affiliations:** ^1^ Edinburgh Napier University Edinburgh UK

**Keywords:** inclusive research, intellectual disabilities, medication management, participatory research, prescription medication, self‐management

## Abstract

**Background:**

There is little research that has examined what support strategies are effective to help adults with intellectual disabilities take their prescribed medication correctly. The aim of the study was to gain an understanding of the barriers and supports that contribute to adults with intellectual disabilities self‐managing their prescribed medicines.

**Methods:**

Nine adults with intellectual disabilities and two support staff participated in this two‐phase study. Phase 1 consisted of focus groups and Phase 2 involved participants taking part in simulated real‐world scenarios based on situations discussed during Phase 1 about taking prescription medication.

**Results:**

Three main themes emerged from the findings: knowledge about prescription medication, barriers to taking prescription medication, and facilitators to taking prescription medication.

**Conclusions:**

The findings of the study will help to inform healthcare professionals on how to better support adults with intellectual disabilities to take their prescription medication to ensure better health outcomes.

**Summary**

- Adults with intellectual disabilities who took part in this project had a good understanding of their health conditions and prescribed medication.
- Barriers to taking prescribed medication identified by adults with intellectual disabilities included unclear communication and inaccessible instructions, unexpected visual changes to medication or access issues, forgetting to take medication, and limited formal and informal support. Clear communication, accessible written information, routines and reminders, and formal and informal supports were highlighted as facilitators to taking prescribed medication.
- This information will help health professionals to better support the needs of adults with intellectual disabilities when taking prescribed medicines to ensure better health outcomes.
- The research team was co‐led by two citizen researchers with intellectual/developmental disabilities to ensure the voices of people with lived experiences were represented in each stage of the research project.

## 1 Introduction

Being prescribed medication is the most common medical intervention when people access healthcare. Ensuring that individuals receive maximum benefit from the medication they are prescribed is critical to effective healthcare management. However, research suggests that between a third and a half of all prescribed medication is not taken as directed, resulting in disease progression, hospitalisation, and increased cost to the healthcare system as a whole (Healthcare Commission 2007; NICE 2009). There are many reasons for people not to take their prescribed medication correctly. Some personal reasons may be poor understanding, cognitive impairments, vision problems, poor hand coordination, depression, forgetting to take medication, or the medication regime being too complex (Payne 2014; Horne et al. 2005). Research has also found that people may not take their prescribed medication due to their concerns about side effects or their belief that they do not need to take the prescribed medication. Other factors include environmental barriers such as the availability of practical support and difficulties in accessing prescriptions (Payne 2014).

People with intellectual disabilities are living longer with complex physical and mental health needs that require a more complex treatment regime (Haveman et al. 2011), such as requiring multiple prescription medications. For this reason, it is important that we understand how adults with intellectual disabilities self‐manage taking their prescription medications. Due to the health needs commonly associated with having an intellectual disability, people with intellectual disabilities may experience increased medicine use, further complicating medicine‐taking activities. A retrospective study of 37,000 people with intellectual disabilities found that medicine use is 4 times higher for people with intellectual disabilities than that of the general population (Nurminen et al. 2023), and prescriptions of antipsychotic medication are higher in people with intellectual disabilities (Doan et al. 2013). Polypharmacy (when a person is prescribed 5 or more medications) is common for people with intellectual disabilities, resulting in medication not being taken properly, increased risk of harmful drug events including drug/drug interactions, increased hospitalisation, and medication side effects (Erickson et al. 2020; McMahon et al. 2022; Lonchampt et al. 2021). Individuals with intellectual disabilities can be at risk of poor medication knowledge due to lack of accessible information, ineffective communication from healthcare professionals, and cognitive difficulties associated with their intellectual disability (Smith et al. 2019).

The difficulty in taking prescribed medicines goes beyond the act of taking tablets (pills). It involves understanding medical instructions, managing side effects, establishing complex regimes, and effective and meaningful communication with health providers. Thus, supporting people with intellectual disabilities to optimise taking their medication correctly is a complex process that involves physical, psychological, and psychosocial factors and is influenced by the nature of the medical condition, the complexity of the medication routines, and personal characteristics. A study by Paterson et al. (2020) compared medicines taking in people with and without intellectual disabilities, and while the frequency of medicines taking was similar in both groups, barriers such as identifying side effects, motivation to take medicines, and absence of social support were evident.

For these reasons, it is important that we better understand the barriers and supports that adults with intellectual disabilities experience when taking their prescribed medicines. However, little research has been done examining how adults with intellectual disabilities self‐manage taking their prescribed medication. Past research has examined interventions and strategies of how to improve people with intellectual disabilities to take their prescribed medication correctly; however, these studies targeted clinicians and carers. For example, adults with intellectual disabilities living in family homes and semi‐independent settings were found to be six and four times more likely to not take their antiepileptic medication compared to individuals living in group homes (Hom et al. 2015). Living in a supervised residence and having more frequent contact with primary care providers was also found to increase adherence to anti‐hypertensive medication (Cyrus et al. 2019).

However, there is very little research that has examined the barriers and supports from the direct perspective of adults with intellectual disabilities. The aim of the study is to gain an understanding of the barriers and supports that contribute to adults with intellectual disabilities self‐managing their prescribed medicines. The study used participatory research principles to ensure the meaningful involvement of people with intellectual disabilities. By shedding light on this important aspect of healthcare, we hope to contribute positively to the overall health and wellbeing of adults with intellectual disabilities, promoting inclusivity and equitable access to quality healthcare.

## 2 Methods

### 2.1 Research Team

This participatory research project included two paid citizen researchers with intellectual/developmental disabilities as part of the research team. The citizen researchers took a leading role at various stages of this project, such as facilitating the focus group sessions, leading group discussions, analysing and interpreting data, and co‐writing this article. Including people with intellectual/developmental disabilities as part of the research team ensures that the knowledge being created for people with intellectual disabilities is being led by people with intellectual disabilities (Spassiani et al. 2017). The citizen researchers provided an advocacy perspective to the study due to their lived experience and were able to ensure the project design, focus group questions/scenarios, and the analysis of the data was interpreted through a disability lens.

### 2.2 Participants and Recruitment

Participants were recruited via disability organisations working with adults with intellectual disabilities and from existing service user groups known to the research team. Nine adults with intellectual disabilities and two support staff agreed to participate. Ethical approval was obtained from Edinburgh Napier University (#2859622). No financial incentive was offered to participate in this study.

### 2.3 Data Collection

Data was collected in two phases. Phase 1 consisted of two focus groups (*n* = 6; *n* = 5) where adults with intellectual disabilities and support staff were invited to share their experiences of taking prescribed medication, what makes it easy or hard to take medication, and general understanding of their prescribed medication. Phase 2 consisted of simulated (role playing) real‐world scenarios with nursing students at the Clinical Skills Centre at Edinburgh Napier University. The scenarios were based on situations discussed during the focus groups (Part 1) about how medicines taking can be improved for adults with intellectual disabilities. This was an interactive and reflective process where feedback was discussed, and scenarios were adapted to include feedback from adults with intellectual disabilities to gain a better understanding of how to support adults with intellectual disabilities take their prescribed medication correctly.

The first scenario was about an adult with intellectual disabilities being prescribed new inhalers to manage their asthma, and the second scenario was about a new support worker assisting an adult with intellectual disabilities with taking their medication. Participants' feedback on the interactions and the way information was communicated was used to encourage further discussions on medicine self‐management and to modify the scenarios to reflect the comments on what would be helpful for adults with intellectual disabilities in those situations. Six adults with intellectual disabilities took part in phase 2.

Both phases were co‐facilitated by two citizen researchers and the research team, and all sessions were voice‐recorded, transcribed, and anonymised.

### 2.4 Data Analysis

The focus groups and discussions during the real‐life scenarios were analysed using thematic analysis (Patton 2002). The data was coded into themes, and two members of the research team collaborated with the citizen researchers to check for their accuracy, develop final themes, and ensure agreement on their interpretation.

## 3 Results

The analysis found three main themes: (1) knowledge about prescription medication, (2) barriers to taking prescription medication, and (3) supports to taking prescription medication. These themes will be discussed below. From the analysis, we identified seven barriers and four facilitators to taking prescribed medication by people with intellectual disabilities (Table 1).

**TABLE 1:** Barriers and supports to taking prescribed medication by people with intellectual disabilities.

Barriers	Supports
Jagon and unclear communication Inaccessible written information and instructions Issues with accessing prescription medication Changes to regular medication routines Unexpected visual changes to the medication or packaging or blister packs Forgetting to take prescription medication and side effects Limited formal and informal support	Health professionals to communicate clearly Accessible written information Routine, organising and reminders Formal and informal support

### 3.1 Theme 1: Knowledge About Prescription Medication

Overall, adults with intellectual disabilities in our study had a good understanding of their health conditions and were able to explain their diagnoses, the names of the medication they were taking, the reasons for taking them, and describe their medication routines. We spoke to people with a variety of health conditions, including diabetes, epilepsy, cancer, depression, anxiety, muscular problems (spasms), asthma, sleep apnoea, and allergies.

Adults with intellectual disabilities showed knowledge of the potential side effects of medication and consequences of not following their medication regime. For example, two individuals with intellectual disabilities described being hospitalised due to not taking their medication correctly, including missing tablets and the incorrect use of an inhaler. However, all participants felt it was important to take their prescribed medication and expressed motivation to take it correctly.

### 3.2 Theme 2: Barriers to Taking Prescription Medication

#### 3.2.1 Jargon and Unclear Communication

Adults with intellectual disabilities and support staff talked about how medical professionals often communicate using jargon and medical terms rather than plain language. They described situations when doctors and nurses explained new diagnoses, medical tests, and examinations in a way that was inaccessible and left them feeling confused, stressed, frustrated, and struggling to retain the information. For example, one adult with intellectual disability said:

[The doctor] explained that I'm classed as a category “pre‐diabetic”. I knew the word “diabetic”, what that meant cause there is a history of that in my dad's side of the family. But she didn't really fully explain what it was. Participant 2

Poor communication can leave people with intellectual disabilities unclear about how or when to take their prescribed medication or use medical devices such as inhalers. This left them feeling worried and anxious about the potential negative consequences of taking their medication incorrectly. For example, an adult with intellectual disabilities stated:

I once was at the doctor, a few years back and the doctor was trying to explain to me about how to take this tablet [pill] that he was giving me. And I kept asking questions about it because I was all muddled up and wasn't sure what I was doing or when I was to take the tablet [pill]. Participant 9

#### 3.2.2 Changes to Regular Medication Routines

Participants talked about the worry and difficulties they experience when new medication is added to their already complex routines, particularly when no detailed instructions are given by the prescribing professional on how to adjust their regular routines. An adult with intellectual disabilities said:

The two [tablets/pills] I was on I was kinda managing but now that I'm taking more and different ones, it is quite hard. And trying to remember all the different times as well, that's the thing I'm struggling with. Cause like I forgot to take my medication last night. Participant 2

Participants spoke about how it can be challenging or frustrating for adults with intellectual disabilities to work out the correct way to incorporate short‐term prescribed medication, such as antibiotics in their regular routines. These usually come separate from their regular medication and often include their own directions for use and other information, such as potential interactions with other medication.

#### 3.2.3 Inaccessible Written Information and Instructions

The language used on the medication packaging and prescription labels was often seen as inaccessible for adults with intellectual disabilities. The written instructions and directions for use can be confusing and misleading, and some might not understand what is meant by phrases such as “three times a day”, or “with food”, or “on an empty stomach”. One adult with intellectual disabilities explained:

(…) It will be like “take one sachet three times a day”. Which confuses me because then it's like (…) is it saying three times a day like Monday, Tuesday, Wednesday, or does it mean you are taking it three times on a Saturday and then three times on a Sunday to complete the two‐day cycle? Participant 7

Some participants reported using “blister pack” boxes, which contain a weekly supply of medication pre‐organised by the pharmacy into sealed compartments with pills to be taken at a particular time of the day. While these were perceived as useful medication aids, participants also highlighted inaccessible design of some blister packs. This included small writing, confusing layout, and minimal information on the medication it contains. One adult with intellectual disabilities said “The blister packs that I had, the writing on that was ok but now the blister pack that I get is different (…) the writing on it is far too small, hard to understand” *(Participant 9)*.

#### 3.2.4 Unexpected Visual Changes to the Medication or Packaging

Adults with intellectual disabilities and support staff felt that the names of the medications are often too complicated to remember or pronounce. They described relying on the visual appearance of the packaging and tablets to help them identify the medication they are taking. However, it was highlighted that the appearance of the medication, such as its shape, colour, and size or box design can change unexpectedly, with no prior notification from the pharmacy. This can leave adults with intellectual disabilities feeling anxious, confused, and unsure whether they have received the correct medication. For example, two adults with intellectual disabilities describe their experience below:

The medication sometimes changes in tablet form and sizes, depending on if you're in hospital or in your own home (…). So one minute it can be like orange or green (…) and it can be a bit confusing because then I don't know if I'm taking the right dosage or is it a completely different medication. Participant 6

Some weeks it can be the blue tablet and other weeks it's actually a white tablet and it just confuses me because you don't get told about it. It's the same name that's on it but I still phone up the chemist to double check. Participant 9

#### 3.2.5 Forgetting to Take Prescription Medication

Forgetting to take prescription medication was a common theme, and some individuals talked about not knowing what to do when that happens. A support worker highlighted how deciding on a correct course of action for an adult with intellectual disabilities can be very challenging, especially if they only have visiting support or no support. An adult with intellectual disabilities stated:

Before COVID hit sometimes I was forgetting to take my tablets and wasn't taking them. Sometimes I could go a week or two without taking any tablets and that's just because I was forgetting. Participant 9

A support staff member described their experience below:

If [they] do miss it, that's the worrying part (…) Would [they] phone NHS 24 to say I've missed this or would [they] think, oh I'll just take it now (…) or would [they] miss it altogether. It's quite hard for someone with visiting support to make that decision. Staff 1

One adult with intellectual disabilities also talked about considering the medication side effects, such as drowsiness and their plans for the day to determine whether they should take the missed dose the next morning. They explained their experience as:

Because I forgot to take that last night, I thought should I take it today but I thought no, cause (…) there's a high chance I would end up falling asleep and feeling drowsy. Participant 2

#### 3.2.6 Issues With Accessing Prescription Medication

Some adults with intellectual disabilities and support staff described their frustrations at the challenges they experienced when ordering or collecting medication from the pharmacy. These included poor communication between the pharmacy and the GP surgery as well as medication not being ready or only partially ready for collection at the agreed time. This often led to confusion and anxiety as it made organising the medication more difficult and required the individual to return to the pharmacy. One support staff member described their experience below:

And how many times have we gone to the pharmacy and the pharmacist says, I've only got this amount today, you will need to come back in the morning and collect the rest of it. And so for people that maybe can't get out every day or people with a bit of anxiety that can only get their support worker [on certain days this can become a problem]. Staff 1

Other issues included complicated procedures for ordering prescription medication, such as filling out a paper form with the name of all required medication, an online app, or phoning the doctor's office. Individuals talked about their frustrations in how the systems in place are not easy and create barriers to accessing their medication in a timely manner.

#### 3.2.7 Limited Formal and Informal Support

Adults with intellectual disabilities shared stories of the challenges they experienced with accessing formal support, such as being considered ineligible for services or not receiving the support they were entitled to due to staff shortages and other system constraints. As a result, many adults with intellectual disabilities do not have support to ensure they take their prescribed medication consistently and correctly.

There were examples of adults with intellectual disabilities developing informal support strategies with friends, but this was not always a reliable method. One adult with intellectual disabilities described helping a friend with intellectual disabilities organise her medication into an electronic pill box while she was lacking support due to staff shortages:

[My friend] has a carer but she's lacking care now. So I'm kinda going up and helping her. So I helped her make sure that she's got it all in and (…) the correct days that she takes it. Cause she said she wouldn't like to trust herself, just sitting down and doing it herself in case she put one in the wrong day or sometimes she says she can easily put two lots in one day. I think also if you live by yourself, some tablets can look very similar, there's a chance you can mix them up and take the wrong one. Participant 2

### 3.3 Theme 3: Facilitators to Taking Prescription Medication

#### 3.3.1 Health Professionals to Communicate Clearly

Adults with intellectual disabilities found it helpful when healthcare professionals explained information about their medication in a clear, step‐by‐step way and checked in with the individual to make sure they were understanding the information correctly. Understanding why they were being prescribed the medication, how to take it correctly, and the possible side effects was seen as helping them feel more confident and motivated to take their medication.

It was suggested that during the appointment healthcare professionals could help adults with intellectual disabilities create personalised routines and timeframes for taking their medication or incorporating new medication into their schedules. One adult with intellectual disabilities stated:

When the doctor says take that amount of tablets in the morning, once a day or twice a day. I then have to work out the time [on my own] (…) Because if they don't give me the timing, and often they don't, then I have to work that out for myself. So if I had the time [written] on [the prescription label], it would be helpful. Participant 8

It was highlighted as important that health professionals explain the different types of medication and medical devices available as well as their benefits and side effects so adults with intellectual disabilities can make an informed choice about which option is the best and easiest for them to use. One adult with intellectual disabilities explained:

I spoke to my GP and I've also spoke to my nurse about [my inhaler]. And they changed [my inhaler] over to easy click. I don't know why they didn't offer them to me in the first place. It would have been a lot easier. But I think when someone is diagnosed with asthma, they need to be told the different types of inhalers that they can use. Participant 8

Adult with intellectual disabilities and support staff suggested it would be helpful to be notified by the pharmacy about any medication supplier changes, which could result in the packaging and/or the size or colour of the tablet changing. This would reassure adults with intellectual disabilities that they have received the correct medication.

### 3.4 Accessible Written Information

Adults with intellectual disabilities and support staff highlighted the importance of having accessible written information about their medication. This included easy‐read leaflets and detailed prescription labels that clearly described when and how the medication should be taken, and any additional information such as foods or drinks to avoid. One adult with intellectual disabilities described a positive situation when a GP recognised they were confused about how to take their medication and contacted the chemist to provide more information to be included on the label:

I felt [it] was quite good that the doctor had actually realised that I [was] confused and he decided to redo a new prescription and put more detail down so I know what was happening. Participant 9

There were also suggestions for re‐designing the blister packs to make them clearer and more accessible for adults with intellectual disabilities, including using bigger font, including easy‐ready information and leaflets about each medication, and clearer days of the week and times on the individual compartments to reduce the risk of mistakenly taking the wrong dose.

### 3.5 Routines, Organising and Reminders

Adults with intellectual disabilities highlighted the importance of establishing daily routines to manage their medication, such as taking it after breakfast or just before going to bed. Some individuals used reminders and alarms on their phones or other electronic devices to help them remember to take their medication. One adult with intellectual disabilities explained:

I set my alarm on my phone (…). But also Alexa as well. Cause sometimes (…) I can forget. (…) I'm thinking of getting the [electronic pill] box that my friend suggested. It's the one that you can have for different days and different times. You can set the alarm to whenever you need your medication. Participant 2

Having medication organised by the pharmacy into blister packs or pill boxes that can be refilled independently from the original packaging was also seen as helpful to manage medication independently.

### 3.6 Formal and Informal Support

Most of the adults with intellectual disabilities who took part in this project had formal support, such as support workers who assisted them with managing their prescription medication. The two support workers who took part in the focus groups felt that their main role in supporting medication taking included helping with effective communication with health professionals, providing practical support to take medication, repeating medication routines, and reasons for taking medication.

Adults with intellectual disabilities discussed how they rely on support workers to accompany them to any medical appointments and to help understand or repeat any information that the medical professional might give them or clarify any misunderstandings. As one adult with intellectual disabilities explained:

I always find that having a support worker with me when I go to my appointments really helps because then they can reiterate everything (…) And then it helps me to understand why I need to get certain medication or why I need certain treatments. Participant 7

When formal support is not available, participants spoke about relying on friends and family to help them follow their medication routine. This included friends sending each other reminders over the phone or accompanying each other to the pharmacy to collect prescriptions.

## 4 Discussion

The purpose of this study was to gain an understanding of the barriers and supports that contribute to adults with intellectual disabilities self‐managing their prescribed medication. Although this study consisted of a small number of participants, our findings contribute to past research on this topic as it provides the direct perspective of adults with intellectual disabilities, which can help future research and policies to consider when addressing medication self‐management in adults with intellectual disabilities. Furthermore, this study showcases how adults with intellectual/developmental disabilities can be meaningfully included to co‐lead a research project and help create evidence‐based knowledge about the experiences of people with intellectual disabilities. This paper demonstrates to society that people with intellectual/developmental disabilities can think critically about (1) co‐leading research projects and (2) their experience with taking prescribed medicines. Below we will discuss some of the important findings.

### 4.1 Access to Care and Support

Access to appropriate care can support better outcomes for adults with intellectual disabilities. Adults with intellectual disabilities in our study stated that they depended on their support staff to help them take their prescribed medication correctly. Furthermore, individuals stated that when they did not have access to support staff, they relied on family and friends to help them take their medication. The British Medical Association (2022) highlighted that inadequate access to support for vulnerable adults is likely to result in greater levels of unmet need, a reliance on unpaid and informal carers and greater use of the NHS. During focus groups, adults with intellectual disabilities reflected on their experiences of providing support to their friends when formal paid support was unavailable. While there is evidence to suggest that there is a connection between appropriate paid support and a reduction in the use of secondary care services (Spiers et al. 2019), there is also evidence that peer and family support can be effective in supporting positive behaviour change and encouraging people to take their prescribed medications (Paterson et al. 2020; Shahin et al. 2021; Enriquez and Conn 2016). However, neither of these studies mentions the inclusion of people with intellectual disabilities as participants limiting its claims to this population.

### 4.2 Reasonable Adjustments in Communication

The results of this study suggest that adults with intellectual disabilities had an overall good understanding of their medication and medical conditions. Similar findings were reported by Davis et al. (2016), with people with mild intellectual disabilities showing a good understanding of their asthma condition and triggers, the importance of managing it with medication, and the purpose of different medication in reliever and preventer inhalers. However, adults with intellectual disabilities in our study spoke about the difficulty of understanding how to take their medication due to health professionals using words they could not understand (jargon). The use of accessible information is a recognised and effective way to ensure that important health information can be understood by people with intellectual disabilities (Chinn and Homeyard 2017). Organisations such as health and social care providers have a duty under The Equality Act 2010 to make reasonable adjustments for people with intellectual disabilities. The use of written accessible information should be used by all health providers when giving medication information to people with intellectual disabilities to maximise the chances of prescription medication being taken correctly to ensure better health outcomes for adults with intellectual disabilities.

### 4.3 Communication Between Physicians, Pharmacists, and Adults With Intellectual Disabilities

Poor communication between healthcare professionals is known to contribute directly to poor patient safety (Daniel and Rossenstein, 2009). Poor relationships and ineffective systems between physicians and pharmacists can contribute directly to poor patient care (Mercer et al. 2020). Past research has shown that community pharmacists can help support people without disabilities to take their prescribed medications correctly (Torres‐Robles et al. 2022). Pharmacists within primary care are uniquely positioned to positively impact the health outcomes of adults with intellectual disabilities who have multiple chronic health conditions. Pharmacists can help reduce pressures on physicians' services through monitoring long‐term medication use and management of pharmacological (medicine) interventions by strengthening communication between pharmacists and physicians (Waszyk‐Nowaczyk et al. 2021).

By improving communication between physicians and pharmacists, adults with intellectual disabilities may be able to feel better supported to take their prescription medication correctly, thus ensuring better health outcomes. Furthermore, pharmacists should have better communication with adults with intellectual disabilities in letting them know when a medication brand has been changed and pointing out any changes in shape/colour/packaging so individuals with intellectual disabilities are aware of any changes to their medication.

### 4.4 Issues With Requesting Repeat Prescriptions

Adults with intellectual disabilities in our study reported having a hard time when requesting their repeat prescriptions. Past research has found that the process of repeat prescriptions is confusing for both people with and without intellectual disabilities and can be a potential reason for overprescribing (Department of Health and Social Care 2021). Governing organisations like NHS England are reviewing their processes of repeat prescribing to develop new guidelines to increase consistency (Royal Pharmaceutical Society 2023). Other countries should do a similar review to help support people with intellectual disabilities and the general population to be able to renew their repeat prescriptions in a more effective manner.

Our study also found that adults with intellectual disabilities reported using online aps to request their repeat prescription as challenging and could lead to frustration of prescriptions not being refilled. Khanlou et al. (2021) found that while there were undoubtedly benefits to people with disabilities using digital technologies, there was a lack of appropriate support in developing the skills in using digital technologies for people with disabilities. In Scotland, the Government has committed to increasing digitalisation of health and social care, suggesting that through better engagement with digital technologies, people's health and social care outcomes can be improved (Scottish Government 2023). However, it is important that these health and social care digital technologies are inclusive and accessible to the needs of adults with intellectual disabilities.

### 4.5 Recommendations

Adults with intellectual disabilities discussed a wide range of supports and barriers that play a role in their being able to effectively manage taking their prescribed medication. From our findings, we have developed 6 key recommendations to support adults with intellectual disabilities in taking their prescribed medication correctly:

1. Health professionals should take time to clearly explain to adults with intellectual disabilities about:
  - why they are being prescribed medication
  - how they need to take their medication
  - any side effects the medication may have on their bodies or with other medications
2. Pharmaceutical companies (companies that make medications) should work alongside people with intellectual disabilities to ensure medication tablets (pills) and packaging are accessible and consistent. For example, not changing the colour or shape of tablets (pills).
3. Pharmacists should be consistent with the brand of medication given for repeat prescriptions to avoid confusion. If a new medication brand needs to be given, the pharmacist should clearly communicate this to the adult with intellectual disabilities, highlighting the change in packaging and tablet (pill) colour/shape.
4. Primary care doctors and pharmacists should have clear communication pathways to ensure adults with intellectual disabilities are being given the correct medication and that medications are not having negative interactions with each other.
5. Medication packaging should clearly state how and when medication is supposed to be taken to avoid confusion and risk medication being taken incorrectly.
6. Enable support staff to provide adults with intellectual disabilities support in how to manage taking their prescribed medicines correctly.

## Ethics Statement

Ethical approval was obtained from the Edinburgh Napier University (#2859622).

## Conflicts of Interest

The authors declare no conflicts of interest.

## Data Availability

The data that support the findings of this study are available on request from the corresponding author. The data are not publicly available due to privacy or ethical restrictions.
